# Introducing the medical bioinformatics in *Journal of Translational Medicine*

**DOI:** 10.1186/1479-5876-10-202

**Published:** 2012-09-26

**Authors:** Samir K Brahmachari

**Affiliations:** 1 CSIR-Institute of Genomics and Integrative Biology, Mall Road, New Delhi, 110007, India; 2 CSIR- Open Source Drug Discovery (OSDD) unit, Anusandhan Bhavan, 2 Rafi Marg, New Delhi, 110001, India; 3 Council of Scientific and Industrial Research (CSIR), Anusandhan Bhavan, 2 Rafi Marg, New Delhi, 110001, India

## Abstract

The explosion of genome sequencing data along with genotype to phenotype correlation studies has created data deluge in the area of biomedical sciences. The aim of the Medical bioinformatics section is to aid the development and maturation of the field by providing a platform for the translation of these datasets into useful clinical applications. The increase in computing capabilities and availability of different data from advanced technologies will allow researchers to build System Biology models of various diseases in order to efficiently develop new therapeutic interventions and reduce the current prohibitively large costs of drug discovery.

The section welcomes studies on the development of Biomedical Informatics for translational medicine and clinical applications, including tools, methodologies and data integration.

## 

Launched with the promise of improving insights into human health and disease, the Human Genome Project a decade after its completion has revealed a wealth of information. The advent of next generation sequencing technologies and other high throughput measurements of ‘omics’ data, along with clinical phenotype association studies, have created a data deluge. However, explosive growth in biomedical data generation has not yet translated to proportionate increases in clinical returns. The announcement of the X-prize in genomics for sequencing 100 centenarians within 30 days at less than $1,000 per genome, and with an error rate less than one in a million base pairs, is expected to herald a new era, where whole genome sequencing will become routine clinical practice for diagnosis and prognosis for personalized healthcare [1]. However, this would only be possible if we are able to capture, curate and analyze clinical data with ‘omics’ datasets using novel informatics tools to establish correlations with high level of confidence. This major transformation that hoped to bridge the gap between researchers and clinicians will primarily be driven by transformation of silos of biomedical research to an integrative field of intensive data driven discovery.

The new paradigm of scientific discovery arising out of this data exploration is referred to as the “Fourth Paradigm” [2] and combines data capture, theory and computation. This paradigm rests on the power of information technology and advance computing facility to effectively mine semantically linked datasets to derive patterns and predictive models.

‘*Medical Bioinformatics’* holds immense promise in this area by equipping researchers with tools and resources to efficiently capture, curate and analyze the ‘big data’, while allowing clinicians and care-givers access to evidence based insights which could be effectively used for patient-care.

The initial efforts on data collection of genotype-to-phenotype correlation studies have largely been carried under the Genome Wide Association studies (GWAS). Although the studies have contributed immensely towards exploring the genetic basis of complex diseases, many of the insights obtained by these studies could not be optimally extrapolated to clinical settings due to poor predictability of traits exclusively based on genetic markers, baring a few exceptions. Large-scale integrative analyses of these datasets are primarily limited by non-standard phenotype descriptions and limitations with data sharing due to ethical constrains. The field of genotype-to-phenotype association study requires new and effective measures to integrate standardized genotype and phenotype information, so as to enable their easy access and robust analyses by the researchers. The GEN2PHEN, is one of such endeavors that aims to efficiently gather and organize the web-based genetic information that fundamentally impacts human health and disease prognosis [3]. The project has achieved significant success in facilitating holistic viewing of genotype-to-phenotype (G2P) correlations by systematically integrating genetic variation databases of the human and model organisms, and further linking it to other biomedical knowledge sources through genome browser functionality.

While organizing existing datasets still remains a challenge, it is evident that the next generation of biosensors and electronic gadgets which can collect and transmit information on vital signs and clinical parameters coupled with ubiquitous connectivity are poised to be the next big revolution in personalized healthcare, generating voluminous data on individuals. Systematic organization, and mining of this large repository of clinical information, derived from sensors, diagnostic labs and harnessing it for cost-effective and personalized healthcare would be a major challenge in the immediate future and has raised the need for new data handling, integration and web-based community workspace tools. The emerging concept of ‘cloud computing’ has the potential to address these concerns. Cloud computing allows for collection and storage of clinical data in an advanced information technology enabled fashion. Information transmitted through biosensors could be received and stored in a transparent manner and in a standardized electronic format. The information could be archived with additional non-clinical parameters, thus forming the basis of a biomedical database. Such cloud computing will increase the access and the workflow of electronic data to enable accurate analysis towards the discovery of new predictive biomarkers for disease predisposition.

The advancement in techniques for data capture and curation of different types of data will further encourage adoption of an integrated, ‘System Biology’ platform for studying and analyzing these datasets. This approach will enable designing of accurate predictive mathematical models to study biological systems- intracellular networks, cells, organs, and any biological entity—by measuring and integrating genetic, proteomic and metabolic data. With respect to drug discovery, application of this approach will involve utilizing clinical samples from diseased and healthy (normal) individuals to uncover system biology markers and pathways targets, which are indicators of disease and potential targets for therapeutic interventions. Such efficient identification of novel drug target leads thus, has the potential to reduce the current prohibitively large costs of drug discovery by eliminating trial and error methods.

It is also worth mentioning that the big data science of medical bionformatics, which has attracted big attention of funding agencies [4] will engage the expertise of medical scientists, bioinformaticians, computer scientists, and database experts, along with mathematicians and engineering experts in modelling and simulation of biosystems, with an objective of developing novel low-cost therapeutic applications. The Open Source Drug Discovery (OSDD) initiative [5,6] for system level understanding of *Mycobacterium tuberculosis* has created a new paradigm for distributed co-creation through crowd sourcing [7] and the application of social media for data gathering. This is going to be the new frontier of application of bioinformatics in medical research.

The Medical bioinfromatics section in *Journal of Translational Medicine* would aim to promote research in this area by providing a high-quality publishing media for path-breaking and innovative research in the area.

## References

[B1] Baker M Contest to sequence centenarians kicks off Nature 2012 487 417 10.1038/487417a 22836978

[B2] Hey T Tansley S Tolle K The fourth paradigm: data-intensive scientific discovery Microsoft Research 2009

[B3] Thorisson GA Lancaster O Free RC Hastings RK Sarmah P Dash D Brahmachari SK Brookes AJ HGVbaseG2P: a central genetic association database Nucleic Acids Res 2009 37 Database issue D797 802 18948288 10.1093/nar/gkn748 PMC2686551

[B4] Obama administration unveils “big data” initiative Announces $200 million in new r&d investmentshttp://www.whitehouse.gov/sites/default/files/microsites/ostp/big_data_press_release.pdf

[B5] Singh S India takes an open source approach to drug discovery Cell 2008 133 201 3 10.1016/j.cell.2008.04.003 18423188

[B6] Bagla P CrowdSourcing Drug Discovery Science 2012 335 909 10.1126/science.335.6071.909 22362985

[B7] Vashisht Crowd Sourcing a new paradigm for Interactome Driven Drug Target Identification in Mycobacterium tuberculosis PLoS One 2012 7 7 e39808 10.1371/journal.pone.0039808 22808064 PMC3395720

